# The Effect of SPECTROM Training on Support Staff Knowledge of Psychotropic Medicine and Attitude Towards Behaviours That Challenge in Adults With Intellectual Disabilities to Help Implement the STOMP Initiative

**DOI:** 10.1111/jir.13236

**Published:** 2025-03-26

**Authors:** B. Limbu, S. Deb, J. Bradshaw, V. Cooper

**Affiliations:** ^1^ Department of Brain Sciences, Faculty of Medicine Imperial College London London UK; ^2^ The Tizard Centre University of Kent Canterbury UK; ^3^ The Challenging Behaviour Foundation The Old Courthouse Chatham UK

**Keywords:** adults with intellectual disabilities, attitude to behaviours that challenge, psychotropic medicine knowledge, RCT, SPECTROM, STOMP, support staff training

## Abstract

**Background:**

Overmedication of adults with intellectual (learning) disabilities, particularly the off‐licence use of psychotropic medicines for behaviours that challenge in the absence of a psychiatric disorder, is a major public health concern and an example of health inequalities. In the United Kingdom, we coproduced online training resources backed up by face‐to‐face training for support staff (direct care workers) called SPECTROM involving all stakeholders, including adults with intellectual disabilities and their families, to help reduce the overmedication and implement NHS England's STOMP initiative.

**Method:**

In a feasibility cluster randomised controlled trial, we trained service managers and support staff using two core modules of SPECTROM, namely, (a) Medicine/STOMP and (b) Alternatives to Medicine (ATM) using an online platform. These two core modules introduced 12 other modules and other online resources. We assessed trainees' knowledge of psychotropic medicines using the Psychotropic Knowledge Questionnaire‐Revised (PKQ‐R) and their attitude to behaviours that challenge using the Management of Aggression and Violence Attitude Scale‐Revised‐Intellectual Disabilities (MAVAS‐R‐ID) using a pre–post training design.

**Results:**

The research team delivered SPECTROM training to 18 service managers and 122 support staff. Of the 140 trainees, 126 completed PKQ‐R at baseline before and within 4 weeks after the training. There was a post‐training improvement in PKQ‐R scores in 42 of the 43 questions (97.7%), 22 of which were statistically significant differences (*p* < 0.001). The MAVAS‐R‐ID was completed at baseline and within 4 weeks of training by 125 trainees. The MAVAS‐R‐ID total score showed statistically significant post‐training improvements (*p* < 0.01). Individual domain score analysis showed a statistically significant improvement in one of the five domains related to attitude regarding the use of medicine for behaviours that challenge.

**Conclusions:**

The SPECTROM training seems to improve staff knowledge of psychotropic medicine, at least in the short‐term, and attitude towards behaviours that challenge, particularly concerning the use of psychotropic medicine.

## 1 Introduction

Overmedication of people with intellectual (learning) disabilities, particularly the off‐licence use of psychotropic medicines for challenging behaviours (also known as behaviours that challenge, behaviours of concern and behaviours of distress), such as verbal aggression, physical aggression to others or property, or self‐injury in the absence of a psychiatric diagnosis, is a major public health concern (Deb 2024). To address this issue, NHS England (NHSE), UK embarked on a long‐term NHS plan, ‘STopping Over‐Medication of People with learning disabilities, autism or both (STOMP)’ 6 years ago (Branford et al. 2018). Despite STOMP and national (NICE 2015) and international guidelines (Deb et al. 2009) recommending the use of non‐pharmacological interventions such as positive behaviour support (PBS) (Gore et al. 2022) first for challenging behaviour, the rate of psychotropic use has not changed much in the last three decades (Deb and Fraser 1994). Around half (49%–63%) of adults with intellectual disabilities receive psychotropics (Deb 2024), primarily antipsychotics (24%–32%), compared with < 1% in the general population who receive antipsychotics (Marston et al. 2014), whereas the prevalence of psychosis is 2%–4% in adults with intellectual disabilities (Deb et al. 2022) for which the antipsychotics are primarily licenced. Long‐term antipsychotic use increases the risk of side effects such as sedation, constipation, obesity, diabetes and metabolic syndrome, which can impair a person's quality of life (QoL) and lead to hospitalisation and reduced life expectancy (de Kuijper et al. 2013; Zhou et al. 2019; Sun et al. 2023).

As support staff (direct care workers) ask for medicine to control challenging behaviour in the first place and are apprehensive to consider withdrawal of psychotropic medicine, training them to build their knowledge of psychotropics, skilling them up to address challenging behaviour without using medicine, and giving them confidence to consider psychotropic withdrawal could be a useful strategy towards the reduction of the overmedication and implementation of the STOMP objectives, although staff themselves will not change the prescription.

In the United Kingdom, we have developed SPECTROM (a Short‐term Psychoeducation for caregivers to help Reduce the Overmedication of people with intellectual disabilities) (https://spectrom.wixsite.com/project) training programme for support staff to address these issues. SPECTROM was a coproduction involving all relevant stakeholders, including adults with intellectual disabilities and their families. We recently conducted a parallel design feasibility cluster randomised controlled trial (RCT) in the United Kingdom involving SPECTROM training. The feasibility outcomes are presented in a separate paper (Limbu et al. 2025a). In this brief report, we presented details of one non‐feasibility outcome, namely, the effect of SPECTROM training on the support staff's knowledge of mental health medicines and attitude towards behaviours that challenge.

## 2 Methods

We randomised clusters (community homes and supported living accommodations) in a 2:1 ratio into the SPECTROM training and training as usual (TAU) groups. For each cluster in the TAU group, there were two clusters in the intervention group, where the staff and service/home managers (participants) received the SPECTROM training. Participants in both groups received their organisation's standard training. The study statistician randomly assigned participants (2:1) remotely and blindly using a random number generator (see Limbu et al. 2025b).

### 2.1 Participants

We recruited participants from six national social service provider organisations that support adults with intellectual disabilities in community settings in England and Wales in the United Kingdom. A senior manager in each organisation approached service managers within their respective organisations, who subsequently approached the staff team they supervise for participating in the training. Each service manager was asked to choose a staff team from only one community home/supported living accommodation where at least one adult with intellectual disabilities receives psychotropic medication. This study was approved by The UK West Midlands‐Coventry & Warwickshire Research Ethics Committee (REC reference: 23/WM/0211).

### 2.2 Intervention

SPECTROM is a hybrid/blended learning platform with many online resources backed by face‐to‐face training. As we recruited trainees from a vast geographic area in the United Kingdom, we (SD and BL) delivered the training online. SPECTROM has 14 modules and additional internal and external resources. Two of these 14 modules were used for face‐to‐face training, through which the other modules and resources were introduced. These two modules are core modules (a) Medicine/STOMP and (b) Alternatives to medication (ATM). Unlike other training, SPECTROM is designed not for one‐off training/learning but for life‐long learning by referring to online resources to aid person‐centred care planning.

The Medicine core module covers (a) different psychotropic medicines, their indications, when they could and should not be used, their side effects and monitoring requirements, (b) a discussion about the concern about overmedication and lack of scientific evidence to support the use of psychotropic medicines for challenging behaviour in the absence of a psychiatric disorder, (c) STAR (STOMP Action planning Review) review which provides checklist and tools for staff to conduct their own in‐house staff‐led medicine review, in preparation for a formal medical review by the prescribers, and (d) psychotropic withdrawal, their impact on the person's quality of life, the reasons for withdrawal side effects and how to prepare for this. In session 2, through case vignettes, the good practice points learned in session 1 are reiterated.

The Alternatives to Medicine module is based on the PBS principles, although the approach differs from the traditional PBS training. This module covers the reasons for and the assessment of challenging behaviour, including medical, psychological, psychiatric, environmental and societal causes. The emphasis is on skilling up the staff team to conduct their own assessment of the reasons for the challenging behaviour. We developed the CATS (Comprehensive Assessment of Triggers for Behaviours of Concern Scale) (Limbu et al. 2021) to aid the assessment process. This module discusses how the staff's own attitude towards the behaviour and the person behind the behaviour may impact challenging behaviour and the person's quality of life. This module also covers effective engagement with the person with intellectual disabilities the staff support. We have developed accessible leaflets on 31 commonly used psychotropic medicines for adults with intellectual disabilities to facilitate shared decision‐making with the person with intellectual disabilities and their families. This module also covers different aspects of the ‘Capable environments’ (McGill et al. 2020) in detail.

Several handouts are given to the trainees during the training to facilitate participation in discussions and encourage further activity‐based homework. Other modules cover physical disorders, psychiatric disorders, autism and ADHD.

The training aims to empower, inform, and equip support staff with the knowledge and skills to understand the use of psychotropic medicines, challenging behaviour, and the person behind the behaviour, and to manage their own psychological responses to behaviour through better self‐reflection. The trainees were warned not to change the medication without the prescriber's advice.

Each core module was delivered over four hours with many breaks. However, the trainee feedback revealed that they would prefer each core module to be delivered over a longer period or in divided sessions.

### 2.3 Outcome Measures

Trainees' knowledge of psychotropic medication was assessed with the Psychotropic Knowledge Questionnaire‐Revised (PKQ‐R) (Deb et al. 2021), which was adapted from the Psychotropic Knowledge Questionnaire used in a Dutch study (de Kuijper and van der Putten 2017). The PKQ‐R has 43 questions/items on psychotropic medicines, including their side effects and monitoring requirements. We have amalgamated some items to reduce the total number of items to analyse to mitigate against the Type II error. We have amalgamated questions 16–21 on the ‘side effects of risperidone’ under one heading for scoring purposes. Similarly, we amalgamated questions 22–27 under ‘side effects of sodium valproate’ and 28–33 under ‘side effects of citalopram’ and 34–38 for monitoring requirements for risperidone and 39–43 for the monitoring requirements of lithium under one heading for each. This reduced the total number of items for analysis to 20. Each question is answered as ‘yes’, ‘no’ or ‘do not know’ (see supplementary material). Each right answer was scored as ‘1’, and each wrong answer and the ‘do not know’ answer were rated as ‘0’. Therefore, the total score for all questions could span from 0 to 43 for the all‐item analysis or 0–20 for the amalgamated version. The scale showed moderate to good internal consistency (Cronbach's alpha: 0.58–0.62) and test re‐test reliability (Cohen's kappa: 0.57) (Wilson et al. 2023).

As the original Management of Aggression and Violence Attitude Scale (MAVAS) (Duxbury 2003) was designed to assess the attitude of nursing staff working within in‐patient psychiatric units in the United Kingdom, we adapted this to create MAVAS‐Revised‐Intellectual Disabilities (MAVAS‐R‐ID) to make them relevant to staff working with adults with intellectual disabilities in community settings (Deb et al. 2021). We used the MAVAS‐R‐ID to assess the trainee's attitude to challenging behaviour. The MAVAS‐R‐ID has 22 questions divided into five domains related to attitude to challenging behaviour: (1) internal causative factors, (2) external causative factors, (3) situational/interactional causative factors, (4) management‐medication, and (5) management‐non‐medicinal. The original MAVAS development reported good validity and a Pearson's *r* reliability coefficient of 0.89, and factor analysis identified four factors with eigenvalues of 1.8 or above (Duxbury 2003). The MAVAS‐R‐ID was scored using a 5‐point Likert scale (*strongly agree* to *strongly disagree*) with a maximum score of 120 (see Data S1). The higher scores indicated a more positive staff attitude towards challenging behaviour and the person with intellectual disabilities. A higher score on the rate of ‘disagreement’ indicates a better outcome for domains 1 and 4, and a higher score on the rate of ‘agreement, indicates improvements in domains 2, 3 and 5.

The trainees completed both PKQ‐R and MAVAS‐R‐ID just before and within 4 sweeks of post‐training. The researcher (B.L.) collected the questionnaire data from the trainees via email.

### 2.4 Data Analysis

The pre‐and post‐training scores of PKQ‐R and MAVAS‐R‐ID were analysed using the Wilcoxon signed‐rank paired test as the data were not normally distributed. The total pre‐ and post‐training scores were compared. Additionally, scores of individual questions for PKQ‐R (*n* = 43), amalgamated questions (*n* = 20), and five individual domains for MAVAS‐R‐ID were compared. To mitigate against a Type II error, a Bonferroni adjustment was conducted for the item‐level analysis in PKQ‐R (*p* = 0.0025) and domain‐level analysis for MAVAS‐R‐ID (*p* = 0.01).

## 3 Patient and Public Involvement

The project was coproduced with adults with intellectual disabilities and their families, who were involved throughout the project. A parent (VC) was a project group member. The Tizard Centre Learning Disability Advisory Group (LDAG) provided advice in each stage of the project through their facilitator, a speech and language therapy professor and a project group member (J.B.). The group was constructive in advising on the development of an accessible participant information sheet (PIS), consent forms (CF), and regular newsletters that were posted on the SPECTROM PDP website. A clinical director and head of education in a large provider organisation was also a project team member. One parent not in the project group also sat on the project steering committee.

## 4 Results

Ultimately, 26 community homes were randomised to the SPECTROM training and 13 to the control arm. Eventually, 140 support staff and service managers received training. This included 18 service managers and 122 support staff (86%) out of 142 who consented to participate and received SPECTROM training. Their demographic characteristics are presented in Table 1.

**TABLE 1:** Demographic data of managers and support staff randomised to SPECTROM arm.

Total number of managers randomised to SPECTROM arm (*n*) = 26			*n* response
Gender	Male	4 (25%)	16
	Female	12	
Age	Mean	40 years	16
	Range	27–68 years old	
Highest qualification	Graduate degree or above	8 (53%)	15
	National Vocational Qualification	7	
Number of years of experience	Mean	7 years	16
	Range	2.5–31 years	
Level of staff	Service managers	15 (94%)	16
	PBS practitioner	1	

Total number of support staff randomised to SPECTROM arm (*n*) = 142			*n* response
Gender	Male	56 (45.5%)	123
	Female	67	
Age	Mean	39 years	117
	Range	18–72 years	
Highest qualification	Graduate degree or above	52 (46%)	113
	National Vocational Qualification	47 (41.6%)	
	School leavers	13 (11.5%)	
	Studying	1 (0.9%)	
Number of years of experience	Mean	6 years	113
	Range	1 month to 40 years	
Level of staff	Support Staff	100 (80.6%)	124
	Deputy	9 (7.2%)	
	Senior	15 (12.2%)	

### 4.1 Outcomes

#### 4.1.1 PKQ‐R

**FIGURE 1:**
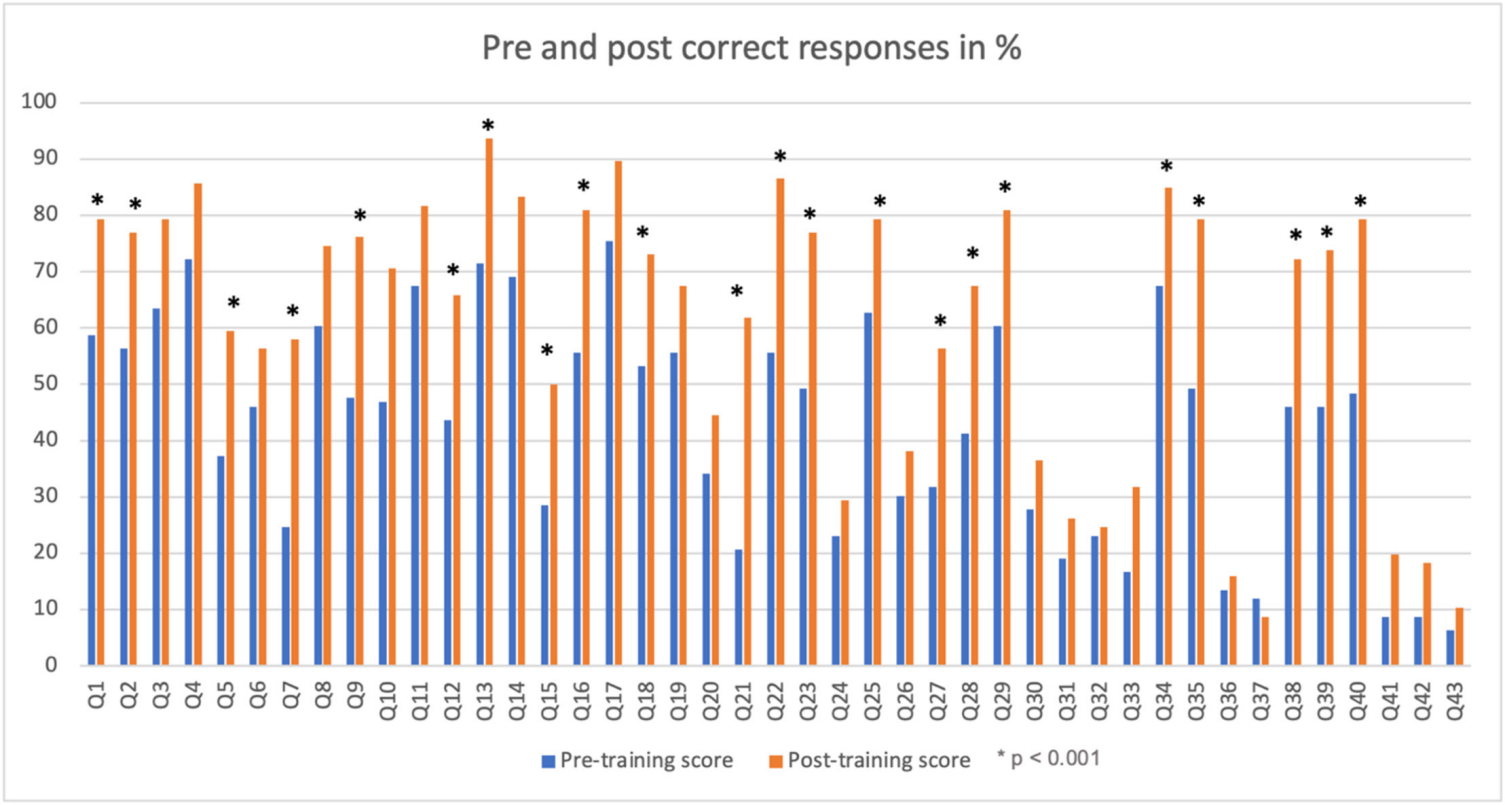
Pre‐ and post‐training PKQ‐R correct answer scores.

Of the 140 trainees, 126 completed the PKQ‐R questionnaire at baseline and four weeks follow‐up. There were less than 10% missing data, which we scored as 0 and not included in the overall score. The pre‐training scores ranged from 0 to 30, while the post‐training scores ranged from 7 to 40. Participants scored significantly more correct answers after the training (median = 26; IQR = 22–30) than before the training (median = 20; IQR = 13–24), (*Z* = −7.95, *p* ≤ 0.001) with a large effect size (*r* = 0.84) (Kerby 2014). Figure 1 shows the percentage of correct answers for each of the 43 questions before and after the training. There were post‐training improvements in scores in 42 (97.7%) of the 43 PKQ‐R items. Of these, post‐training scores on 22 items showed a statistically significant improvement (*p* < 0.001). As for the amalgamated version of PKQ‐R, all 20 items showed improved post‐training scores, of which 14 were statistically significant (*p* < 0.001) (see Table 2).

**TABLE 2:** Summary of correct responses from the amalgamated PKQ‐R questionnaire (*n* = 20).

PKQ items	Question	Baseline %	Post‐training %
Q1***	How many different classes of psychotropic/psychiatric medications are there?	58.7	79.4
Q2***	How often should the staff‐led in‐house medication review be carried out?	56.3	77
Q3	Do you need the opinion of the person with intellectual disabilities about medication use?	63.5	79.4
Q4	Do you need the opinion of parents/family members about medication use?	72.2	85.7
Q5***	Deterioration in some people's behaviour upon withdrawal from psychiatric medication is always related to the withdrawal process	37.3	59.5
Q6	What is an example of risperidone's withdrawal symptom?	46	56.3
Q7***	What is an example of citalopram's withdrawal symptom?	24.6	57.9
Q8	What is an example of a diazepam withdrawal symptom?	60.3	74.6
Q9***	The neuroleptic malignant syndrome is a rare but life‐threatening side‐effect associated with which group/class of psychotropic medication?	47.6	76.2
Q10***	Serotonin syndrome is a rare but serious side‐effect associated with which group/class of psychotropic medication?	46.8	70.6
Q11	Methylphenidate is used for the treatment of …	67.5	81.7
Q12***	Risperidone is an antidepressant drug	43.7	65.9
Q13***	Sertraline is an antidepressant drug	71.4	93.7
Q14	Some antiepileptic drugs are also used for treating behaviours of concern	69	83.3
Q15***	Antipsychotic drugs are very useful in treating the core symptoms of autism	28.6	50
Q16–21***	*Side effects of risperidone* ^a^	49.1	69.6
Q22–27***	*Side effects of sodium valproate* ^a^	42.1	61.1
Q28–33***	*Side effects of citalopram* ^a^	31.3	44.6
Q34–38***	*Check the following regularly when a person is receiving risperidone* ^b^	31.3	43.5
Q39–43***	*Following tests are necessary if a person is receiving lithium* ^b^	19.7	33.6

^a^ Six items combined.

^b^ Five items combined.

*** *p* < 0.001.

### 4.2 MAVAS‐R‐ID

**FIGURE 2:**
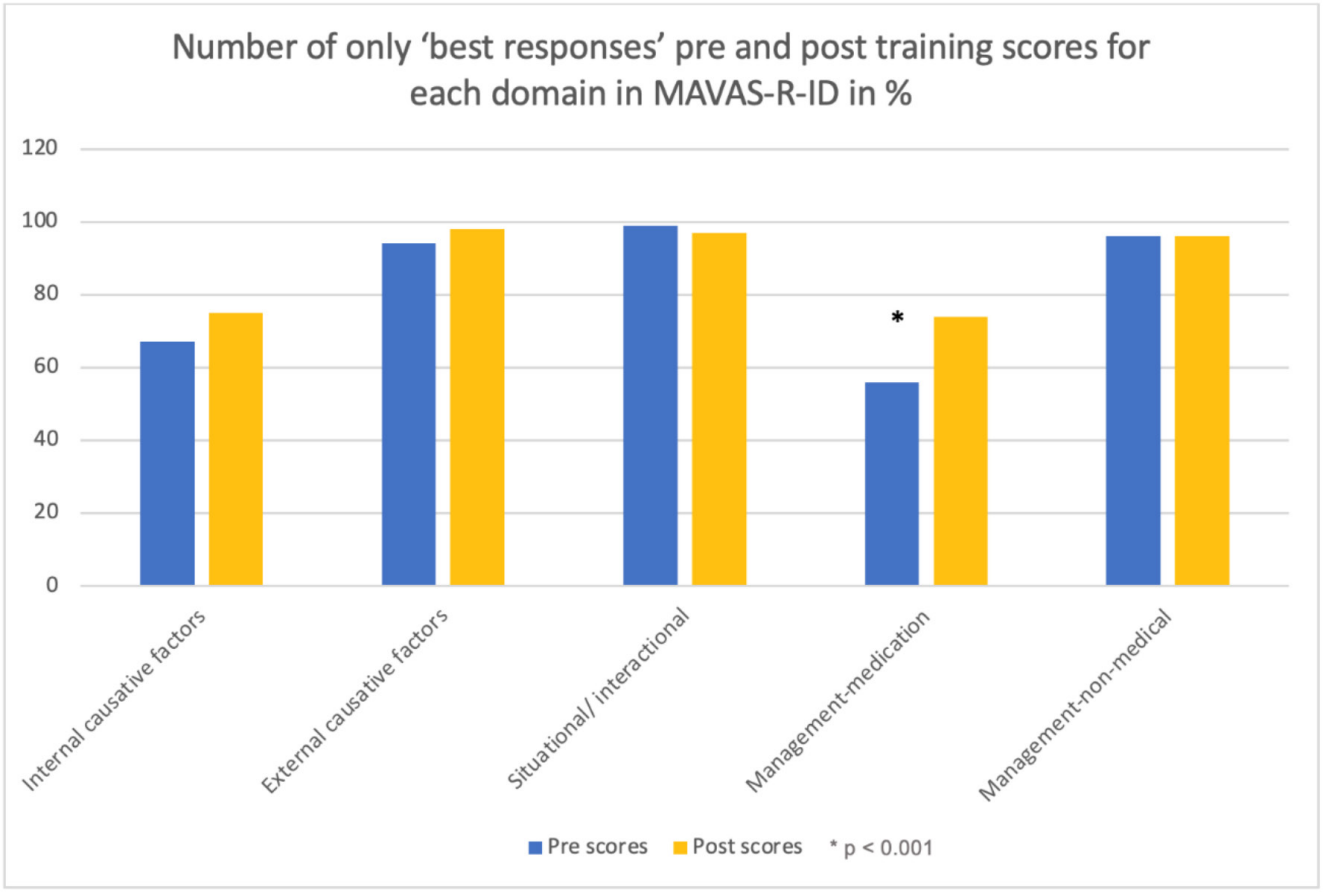
MAVAS‐R‐ID pre‐ and post‐training number of only ‘best responses’ in % for each domain.

**FIGURE 3:**
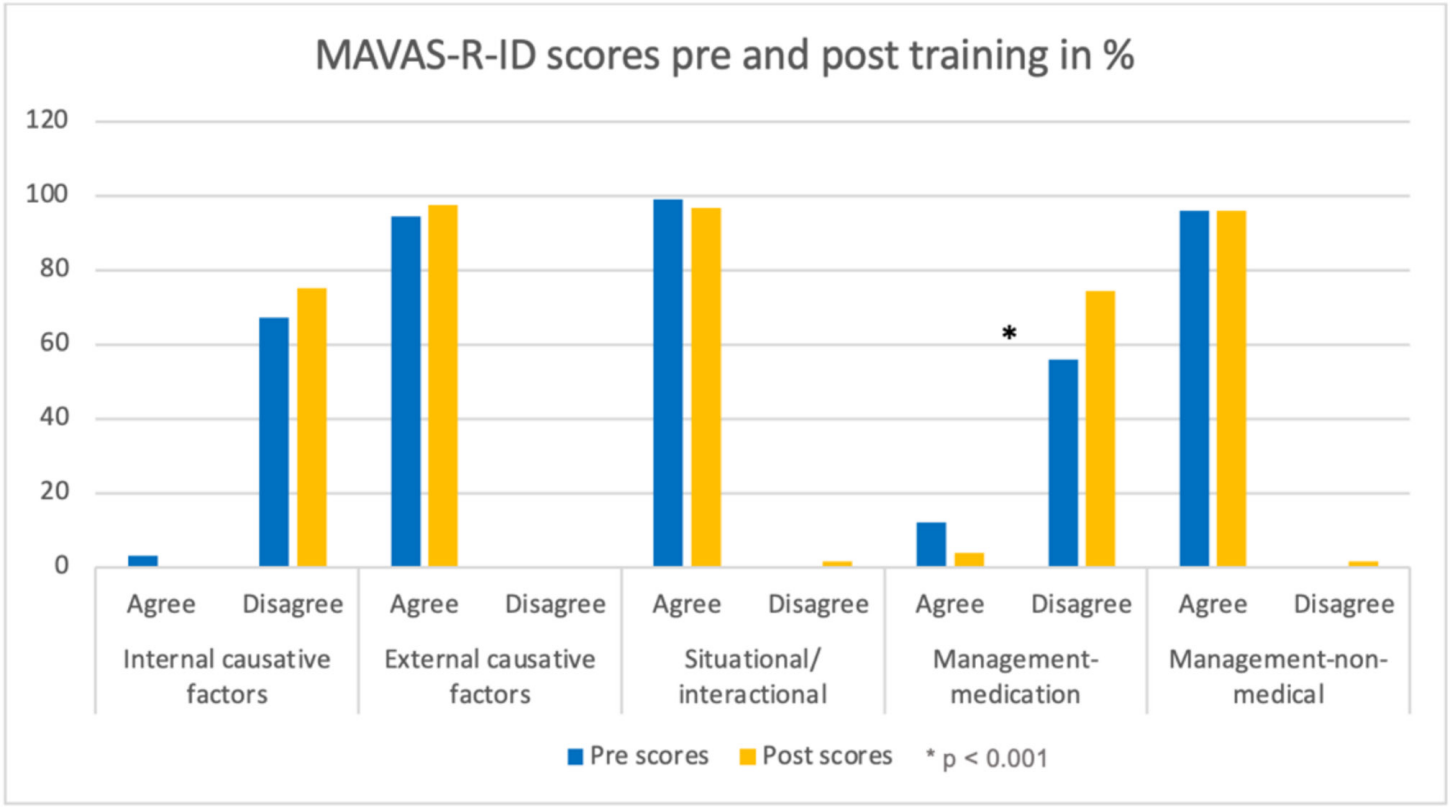
MAVAS‐R‐ID domain‐specific results. A higher ‘disagree’ score for (1) internal causative factors and (4) management medication and a higher ‘agree’ score for (2) external, (3) situational/interactional causative factors, and (5) non‐medical management means improvement in staff attitude towards challenging behaviours. *p ≤ 0.001.

One hundred and twenty‐five trainees completed the MAVAS‐R‐ID questionnaires. There were missing scores for less than 3% of items. The missing score was replaced using the average score. There was a significant improvement in the overall attitude score from before (median = 96; IQR = 89–104) to after (median = 99; IQR = 92–106.5) the training (*Z* = −3.25, *p* = 0.001) with a moderate effect size (*r* = 0.35) (Kerby 2014). Domain analysis revealed that only one domain score (medication‐based management) showed a statistically significant improved post‐training score (median = 3.75; IQR = 3.25–4.25) when compared with the pre‐training score (median = 3.5; IQR = 3–4), (*Z* = −4.46, *p* < 0.01) (see Table 3) (Figure 2). Figure 3 represents the ‘best responses’ for each of the domains. Post‐training increased scores in ‘disagreement’ indicated improvement in domains 1 and 4, of which domain 4 (medication‐based management) score was statistically significant. A post‐training increase in the ‘agreement’ score in domain 2 indicated an improved attitude, but no such trend was observed for domains 3 and 5. However, none of these changes was statistically significant.

**TABLE 3:** Median scores of subdomains in MAVAS‐R‐ID questionnaire at baseline and post‐training, and statistical results with a Bonferroni correction of *p* < 0.01.

Domains	Median at baseline (25th–75th percentile)	Median at post‐training (25th–75th percentile)	Wilcoxon signed‐rank test results
1. Internal causative factors	3.67 (3.17–4.17)	3.83 (3.42–4.25)	*Z* = −2.35, *p* = 0.019
2. External causative factors	4.17 (3.83–4.67)	4.33 (3.92–4.67)	*Z* = −1.11, *p* = 0.27
3. Situational/interactional causative factors	4.25 (4.00–5)	4.50 (4.00–5)	*Z* = −0.80, *p* = 0.43
4. Management‐medication	3.50 (3.00–4)	3.75 (3.25–4.25)	*Z* = −4.46, *p* ≤ 0.01*
5. Management‐non‐medical	4.50 (4.00–5)	4.75 (4.00–5)	*Z* = −1.88, *p* = 0.61

* Statistically significant.

## 5 Discussion

As the support staff are at the forefront of administering medicine and witnessing the side effects in people with intellectual disabilities they support, it is of paramount importance for them to have some basic knowledge of psychotropic medications and their side effects. Previous studies showed that staff had little knowledge of psychotropic medicines and wanted more information on them (Donley et al. 2011; Lalor and Poulson 2013). The SPECTROM training is appropriately designed to address this issue. The current study showed that the training has significantly improved the staff's knowledge of psychotropic medicines. As knowledge is power, this helps to empower staff to engage with the prescribers in an informed discussion about medicines. This finding is similar to what we found in our previous field testing in the United Kingdom (Deb et al. 2021) and a pilot pre‐post study using SPECTROM in Australia (Wilson et al. 2023).

Our previous studies showed that different staff may have different views about using medicine for challenging behaviour. Some felt medicine is a chemical restraint when used for challenging behaviour, but others felt it may be necessary (Deb et al. 2023). Previous studies of staff training showed improvement in knowledge but not necessarily in attitude (Deb and Roberts 2005; Rose and Gallivan 2019). The current study showed improved attitudes towards challenging behaviour, particularly concerning using medicine for challenging behaviour. This finding is similar to what we found in our field testing in the United Kingdom (Deb et al. 2021). However, there is a statistically significant improvement in only one out of five MAVAS‐R‐ID domains (medicine‐management of challenging behaviour).

There may be several reasons for this. The change in attitude may be difficult to measure just using a questionnaire and may only be assessed from the change in the trainee's practice, which will take a long time to change and measure subsequently. It is also possible that trainees did not want to admit their unacceptable attitude in public, causing bias in the pre‐training ratings. However, in the United Kingdom, most large service provider organisations provide PBS training to their staff, which may have helped improve their attitude, and there was little scope to improve after training. Therefore, it is essential to involve staff from smaller organisations in the SPECTROM training.

Although this feasibility study showed that SPECTROM training helped to reduce psychotropic prescribing at 6‐month follow‐up (Deb et al. 2025), without an adequately powered RCT, it is difficult to conclude the training's impact on overmedication. Suppose a more definitive RCT shows a definite effect of SPECTROM training in reducing inappropriate overmedication. In that case, we believe it would be appropriate for the service provider organisations to integrate this training within their existing training portfolio to help implement the NHSE STOMP initiative. However, our feasibility study also showed that although most service provider organisations and their employees were willing and enthusiastic to participate in the training, some were reluctant and quoted lack of capacity as the reason (Limbu et al. 2025c). It was, therefore, felt that many organisations will not implement the training unless there is some leverage used by the regulatory authorities and the paymasters demanding evidence of including a training programme to help implement STOMP. A requirement for evidence‐based training for employees to implement STOMP should also be included in NHS England's Health Improvement Framework to help improve the quality of care for adults with intellectual disabilities.

### 5.1 Limitations

As a feasibility study, we did not have the opportunity to assess knowledge attrition over time. To prevent that, it may be necessary to repeat the training at certain intervals. The lack of long‐term follow‐up also prevented us from assessing how the training affects staff's day‐to‐day practice. For example, does it improve staff communication with the person with intellectual disabilities and facilitate shared decision‐making with the person with intellectual disabilities and their families in designing person‐centred care plans? Although we trained a large number of trainees, we did not calculate the sample size before the study as this is not necessary for a feasibility study like ours. The lack of longer follow‐up data is a limitation of the study. However, we are collecting 6 months of follow‐up data on PKQ‐R and MAVAS‐R‐ID, and the initial analysis so far has shown that the early knowledge gain was maintained at the longer follow‐up. However, as these data were unavailable, we could not include them in the current paper.

## Conflicts of Interest

The authors declare no conflicts of interest.

## Supporting information


**Data S1.** Supporting Information.

## Data Availability

The research team, steering committee and the sponsor's research and development department will have access to data. If appropriate and legal, the data will be made available upon reasonable request and subject to approval from the sponsor and the funder.
