# Supplementary material for: The Effect of SPECTROM Training on Support Staff Knowledge of Psychotropic Medicine and Attitude Towards Behaviours That Challenge in Adults With Intellectual Disabilities to Help Implement the STOMP Initiative
Source: J Intellect Disabil Res. 2025 Mar 26;69(7):630–8. doi: 10.1111/jir.13236 (PMC12198094; doi:10.1111/jir.13236)
Supplement: Supplementary file 1 — Data S1. Supporting Information. [file JIR-69-630-s001.docx]

**Supplementary material (PKQ-R and MAVAS-R-ID)**

**PKQ-R (Psychotropic Knowledge Questionnaire-Revised)**

Date of completion: …………………………………………………………………………………

Initial of the participant: ………………………………………………………………………….

You have been asked to complete these survey questions as you have participated in the SPECTROM training. You will be asked to complete this survey twice, once before the training and again after the training.

When answering the questions, please keep the following points in mind:

1. Your own honest views and beliefs should be reflected in the answers rather than your organisation’s or others’ views.
2. Answer the questions as per your own experience.
3. Do not deliberate on each question for too long. Your first reaction is usually the right one.
4. Please answer these questions as best as you can.
5. We ask that you do not research the answers to these questions if you do not know the answer. We are looking for your baseline knowledge before SPECTROM training and change after the training. Your answers will remain confidential and not be shared with anyone in your workplace.
6. Please circle only one answer for each question you think is right.

| How many different classes of psychotropic/ psychiatric medications are there? | 1-3 | >3 | Don’t know |
| --- | --- | --- | --- |
| How often should the staff-led in-house medication review be carried out? | Every 4-6 months | Once a year | Don’t know |
| Do you need the opinion of the person with intellectual disabilities about medication use? | Yes | No | Don’t know |
| Do you need the opinion of parents/family members about medication use? | Yes | No | Don’t know |
| Deterioration in some people’s behaviour upon withdrawal from psychiatric medication is always related to the withdrawal process. | Yes | No | Don’t know |
| What is an example of risperidone’s withdrawal symptom? | Flu-like symptoms | Parkinsonism (body shakes) | Don’t know |
| What is an example of citalopram’s withdrawal symptom? | Flu-like symptoms | Parkinsonism (body shakes) | Don’t know |
| What is an example of diazepam’s withdrawal symptom? | Parkinsonism (body shakes) | Agitation | Don’t know |
| The neuroleptic malignant syndrome is a rare but life-threatening side-effect associated with which group/class of psychotropic medication? | Antidepressant medications | Antipsychotic medications | Don’t know |
| Serotonin syndrome  is a rare but serious side-effect associated with which group/class of psychotropic medication? | Antidepressant medications | Antipsychotic medications | Don’t know |
| Methylphenidate is used for the treatment of | ADHD | Autism | Don’t know |
| Risperidone is an antidepressant drug. | Yes | No | Don’t know |
| Sertraline is an antidepressant drug. | Yes | No | Don’t know |
| Some antiepileptic drugs are also used for treating behaviours of concern. | Yes | No | Don’t know |
| Antipsychotic drugs are very useful in treating the core symptoms of autism. | Yes | No | Don’t know |
| *Side effects of risperidone* |  |  |  |
| Weight gain. | Yes | No | Don’t know |
| Drowsiness. | Yes | No | Don’t know |
| Muscle stiffness. | Yes | No | Don’t know |
| Shaky hands. | Yes | No | Don’t know |
| Hair loss. | Yes | No | Don’t know |
| Men developing breasts. | Yes | No | Don’t know |
| *Side effects of sodium valproate* |  |  |  |
| Weight gain. | Yes | No | Don’t know |
| May damage the foetus/ unborn baby. | Yes | No | Don’t know |
| Muscle stiffness. | Yes | No | Don’t know |
| Drowsiness. | Yes | No | Don’t know |
| Men developing breasts. | Yes | No | Don’t know |
| Hair loss. | Yes | No | Don’t know |
| *Side effects of* citalopram |  |  |  |
| Sexual dysfunction. | Yes | No | Don’t know |
| Sleep problems. | Yes | No | Don’t know |
| Hair loss. | Yes | No | Don’t know |
| Shaky hands. | Yes | No | Don’t know |
| Life-threatening skin rash. | Yes | No | Don’t know |
| Aggression. | Yes | No | Don’t know |
| *Check the following regularly in a person receiving risperidone.* |  |  |  |
| Weight. | Yes | No | Don’t know |
| Blood sugar level. | Yes | No | Don’t know |
| Thyroid function. | Yes | No | Don’t know |
| Kidney function. | Yes | No | Don’t know |
| Cholesterol level. | Yes | No | Don’t know |
| *Following tests are necessary if a person is receiving lithium.* |  |  |  |
| Thyroid function. | Yes | No | Don’t know |
| Kidney function. | Yes | No | Don’t know |
| Blood sugar level. | Yes | No | Don’t know |
| Cholesterol level. | Yes | No | Don’t know |
| EEG. | Yes | No | Don’t know |

Thank you for your answers.

Professor Shoumitro (Shoumi) Deb, MBBS, FRCPsych, MD, Imperial College London, UK.

Email: [s.deb@imperial.ac.uk](mailto:s.deb@imperial.ac.uk)

**MAVAS-R-ID (Management of Aggression and Violence Attitude Scale-Revised-ID)**

Date of completion: …………………………………………………………………………………

Initial of the participant: ………………………………………………………………………….

You have been asked to complete these survey questions as you have participated in the SPECTROM training. You will be asked to complete this survey twice, once before the training and again after the training

When answering the questions, please keep the following points in mind:

1. Your own honest views and beliefs should be reflected in the answers rather than your organisation’s or others’ views.
2. Answer the questions as per your own experience.
3. Do not deliberate on each question for too long. Your first reaction is usually the right one.
4. Read the questions carefully, as some items are written in reverse order.
5. For each item, tick the box that reflects your own view and experience most honestly.
6. Please ignore the ‘Total score’ and ‘Total sub-score’ sections.

| Items | Strongly agree. | Agree. | Neither agree/ nor disagree. | Disagree. | Strongly disagree. |
| --- | --- | --- | --- | --- | --- |
| **Internal causative factors for the behaviour of concern** |  |  |  |  |  |
| Hearing or vision problems may lead to behaviours of concern. |  |  |  |  |  |
| People with intellectual disabilities show behaviours of concern always because of their disabilities. |  |  |  |  |  |
| People with intellectual disabilities show behaviours of concern always because of mental health problems. |  |  |  |  |  |
| Some genetic disorders are associated with a high level of behaviours of concern. |  |  |  |  |  |
| People with intellectual disabilities always show behaviours of concern deliberately. |  |  |  |  |  |
| People with intellectual disabilities show behaviours of concern because they always want attention. |  |  |  |  |  |
| **Total sub-score** |  |  |  |  |  |
|  |  |  |  |  |  |
| **External causative factors for the behaviour of concern** | Strongly agree. | Agree. | Neither agree/ nor disagree. | Disagree. | Strongly disagree. |
| The environment may play an important part in causing behaviours of concern. |  |  |  |  |  |
| Restrictive environments can contribute towards aggression and behaviours of concern. |  |  |  |  |  |
| Change in a person’s routine never plays a part in causing behaviours of concern. |  |  |  |  |  |
| A low arousal environment may reduce behaviours of concern in some. |  |  |  |  |  |
| Bright lights never lead to behaviours of concern. |  |  |  |  |  |
| Loud noise may lead to behaviours of concern in some people. |  |  |  |  |  |
| **Total sub-score** |  |  |  |  |  |
|  |  |  |  |  |  |
| **Situational/ interactional causative factors for the behaviour of concern** | Strongly agree. | Agree. | Neither agree/ nor disagree. | Disagree. | Strongly disagree. |
| Poor communication between the person with intellectual disabilities and the support staff (direct care worker) never leads to behaviours of concern. |  |  |  |  |  |
| Improved one-to-one relationships between support staff and the person with intellectual disabilities can reduce the incidence of behaviours of concern. |  |  |  |  |  |
| Support staff’s attitude to the person can contribute towards behaviours of concern. |  |  |  |  |  |
| Different situations and environments may contribute toward the expression of aggression by the person with intellectual disabilities. |  |  |  |  |  |
| **Total sub-score** |  |  |  |  |  |
|  |  |  |  |  |  |
| **Management-medication for the behaviour of concern** | Strongly agree. | Agree. | Neither agree/ nor disagree. | Disagree. | Strongly disagree. |
| Medication should always be the first option for addressing aggression and behaviours of concern in a person with intellectual disabilities. |  |  |  |  |  |
| Psychiatric medications should be used more frequently for aggressive behaviour and behaviours of concern in persons with intellectual disabilities. |  |  |  |  |  |
| There is strong scientific evidence that medication reduces aggression in all people with intellectual disabilities. |  |  |  |  |  |
| A large number of medications are currently licenced for treating behaviours of concern in people with intellectual disabilities. |  |  |  |  |  |
| **Total sub-score** |  |  |  |  |  |
|  |  |  |  |  |  |
| **Management-non-medical of the behaviour of concern** | Strongly agree. | Agree. | Neither agree nor disagree. | Disagree. | Strongly disagree. |
| Better communication and engagement with the person are likely to improve behaviours of concern. |  |  |  |  |  |
| Helping the person with skills development is likely to improve behaviours of concern. |  |  |  |  |  |
| Alternatives to using restrictive practices and medications should be used more frequently to help with the behaviour of concern. |  |  |  |  |  |
| The use of de-escalation may be useful in preventing behaviours of concern. |  |  |  |  |  |
| **Total sub-score** |  |  |  |  |  |
|  |  |  |  |  |  |
| **Grand Total score** |  |  |  |  |  |
